# Osteoid osteoma of the base of the coracoid process – A case report

**DOI:** 10.1016/j.ijscr.2019.11.066

**Published:** 2019-12-09

**Authors:** Dalal AlGhoozi, Hamza Gomaa, Rashad Awad, Fahad Alkhalifa

**Affiliations:** Bahrain Defence Force Hospital-Royal Medical Services, Riffa, Bahrain

**Keywords:** Osteoid osteoma, Shoulder, Coracoid, Arthroscopic excision, Case report

## Abstract

**Introduction:**

Osteoid osteomas (OO) are small, well-defined, benign bone tumors of the young that are commonly found in long bones. In rare circumstances, when present elsewhere, they can mimic the symptoms of other pathologies pertaining to that area.

**Presentation of case:**

We present such case in which an "OO" of the coracoid process led to a 2-year delay in diagnosis and management of a young male due to the presenting symptoms resembling those of other shoulder pathologies. Ultimately, the use of advanced imaging modalities proved to be useful in detecting the "OO", which was consequently arthroscopically excised.

**Discussion:**

Appropriate imaging modalities such as CT or MRI often visualize the characteristic nidus more clearly as opposed to an X-ray and should therefore be used early when conservative management of shoulder pain has not been proven to be useful.

**Conclusion:**

A long history of shoulder pain that is not responsive to conservative management in young patients should raise the suspicion of an "OO", and physicians should be encouraged to use early advanced imaging modalities in order to confirm it.

## Highlights

• Osteoid osteomas are benign bone tumors commonly found in long bones.
• Presence of osteoid osteomas elsewhere makes early diagnosis difficult.
• MRI and CT scans help detect osteoid osteomas early.
• Juxta-articular osteoid osteomas are best excised arthroscopically.

## 1 Introduction

Osteoid osteomas (OO) are small, well-defined, benign bone tumors of the young [1,2]. They are commonly found in the shaft of long bones of the lower extremities, and less commonly in flat bones [[2], [3], [4]]. It is classified as an active osteoblast-forming tumor that results in a nidus that can be detected by X-rays [2]. ^“^OO”s cause pain that is partially relieved by NSAIDs and made worse at night [2]. When present in unusual areas, they can mimic the symptoms of other diseases, thus delaying its diagnosis and therefore, appropriate treatment. This is a case report of a patient who presented to the Bahrain Defence Force Hospital – Royal Medical Services with a delayed diagnosis of an “OO” due to its occurrence in the coracoid process of the scapula. The following work has been reported in line with the SCARE 2018 criteria [18].

## 2 Case presentation

**Fig. 1:**
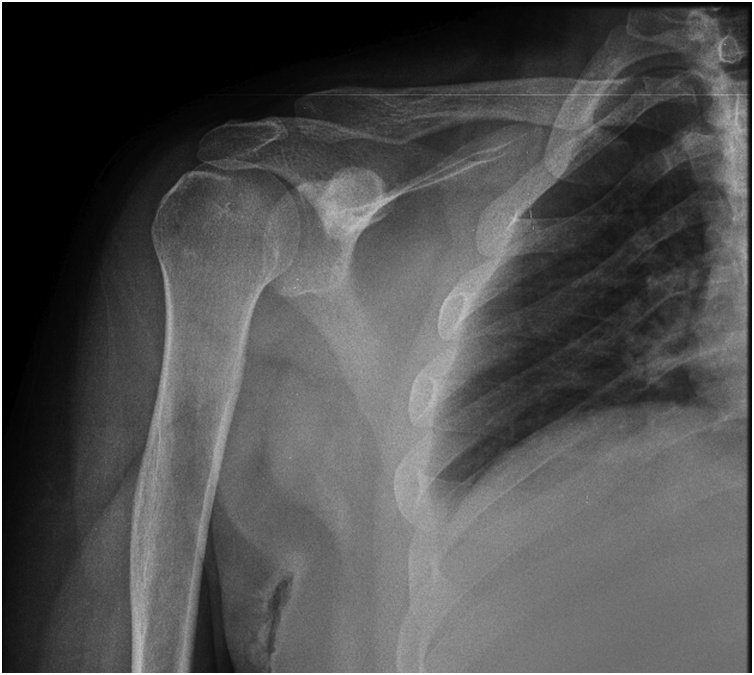
X Ray of the right shoulder.

A 22-year-old male who is not a known case of any medical illness, presented to the clinic following a local hospital referral, with a 2-year history of right shoulder pain. The pain started gradually and increased in severity over time. It was worse at night awakening the patient from sleep. There was no history of trauma or shoulder injury. The patient was referred to physiotherapy with the impression of impingement syndrome based on X Ray and clinical examination findings, yet there was no improvement (Fig. 1). He was then advised for arthroscopic sub acromial decompression following failure of the conservative management.

The pain did not improve after the arthroscopic sub acromial decompression surgery. Following this, the patient started to experience increased pain at rest. Further imaging was ordered including an MRI of the cervical spine and brachial plexus along with a nerve conduction study, all of which were reported to be normal. The patient received a total of 3 intra-articular corticosteroid injections which still did not relieve the pain.

**Fig. 2:**
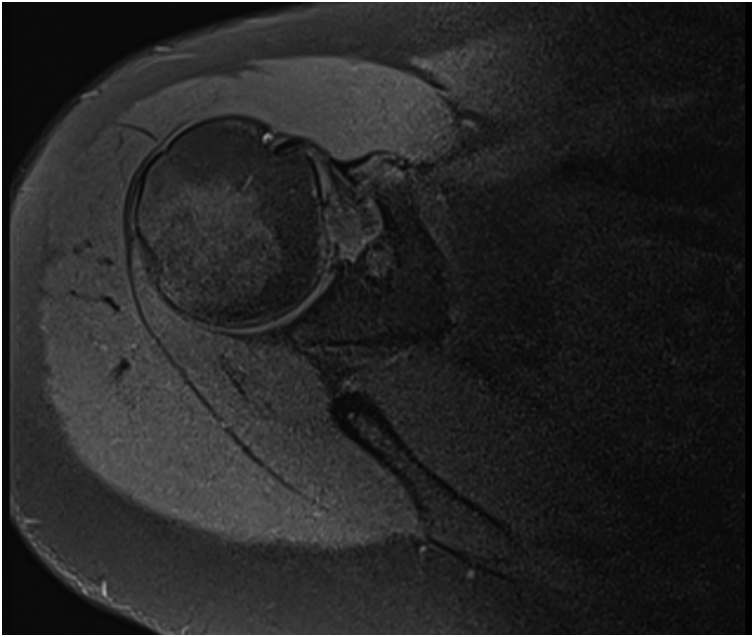
Non-contrast axial MRI of the right shoulder.

**Fig. 3:**
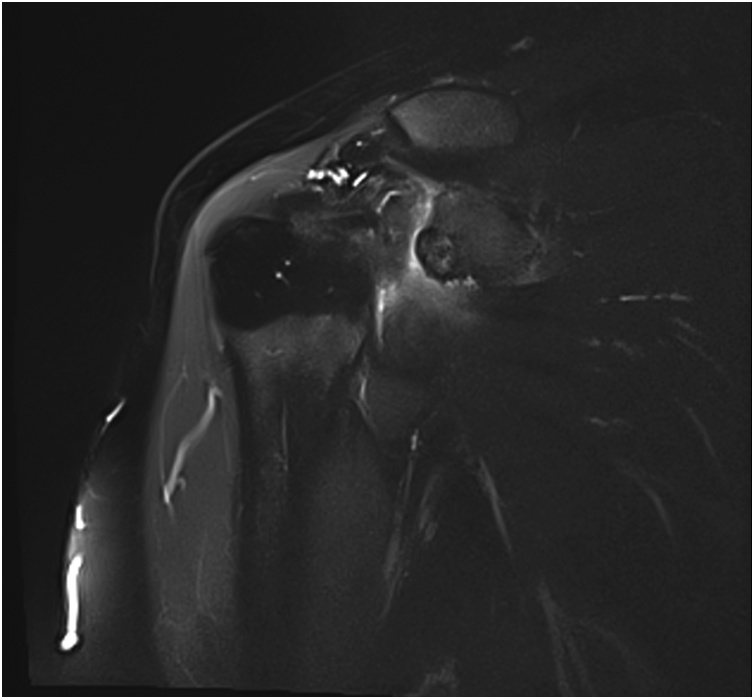
Contrast-enhanced MRI of the right shoulder.

**Fig. 4:**
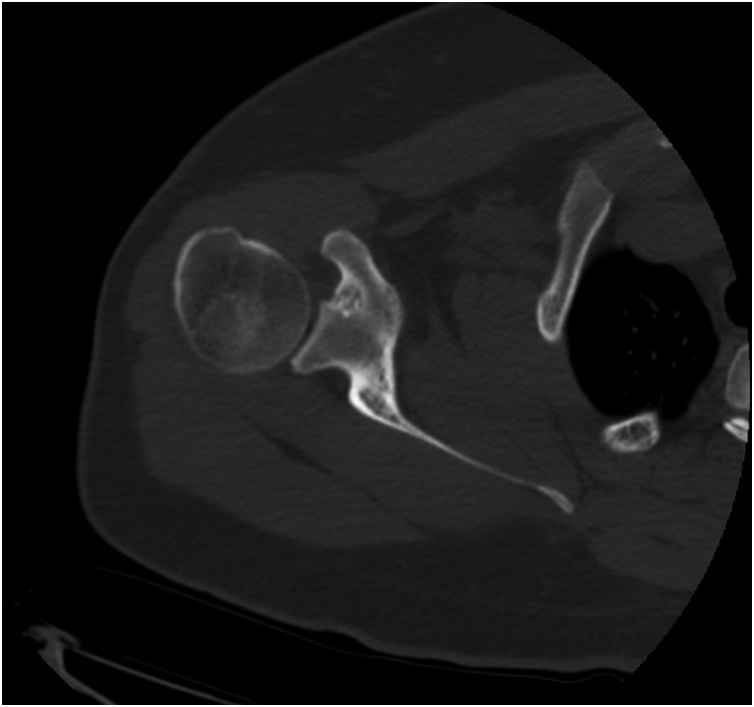
Contrast-ehanced axial CT of the right shoulder.

A non-contrast MRI of the affected shoulder was performed which showed a suspicious lesion at the base of the coracoid. Common coracoid process tumors such as chondrosarcomas, osteoblastomas, and chondroblastomas` [17] were ruled out as subsequent contrast-enhanced MRI and CT scans of the scapula confirmed the presence of a benign “OO” (Fig. 2, Fig. 3, Fig. 4).

After the radiological diagnosis of “OO” of the coracoid process was made by the referring local hospital, the patient was booked for a 2nd arthroscopic shoulder surgery which was performed by a senior consultant of orthopedic surgery. The patient was placed in a beach chair position and image intensifier-guided drilling of the osteoid osteoma lesion was done under general anesthesia. The surgery involved identifying the lesion using a C-arm X-Ray machine followed by the insertion of a guide wire through the lesion and ultimately drilling it with 4.5 mm and 6 mm drills respectively. Arthroscopic shaving and debridement of the lesion followed. The patient was discharged home on the same day in a stable condition with a cuff and collar brace and oral analgesics.

Following surgery, at the 1-week follow up in clinic, the patient reported complete resolution of pain and expressed satisfaction towards the treatment received. The subsequent follow-ups confirmed complete resolution of symptoms and the patient was then advised to mobilize the arm and engage in normal daily activities.

## 3 Discussion

9 cases of osteoid osteomas of the coracoid process of the scapula have been reported in English literature, all of which have been initially misdiagnosed due to its resemblance to shoulder-related pathologies. Although these reported cases involved the typical age group (12–46), typical pain that is made worse at night, and typical partial pain relief by NSAIDs, diagnosis was still delayed (by 3 months-4 years) due to the presence of other factors such as pain radiation [5,9,11] and restricted range of movement [7,10,12]. These factors led to the initial diagnoses to be either: impingement syndrome [8,9,12], arthritis [10], or cervical spine discopathy. [9] Therefore, management was based accordingly with attempts at analgesia [6,8,9,[11], [12], [13]], physiotherapy [9,12], intra-articular steroid injections [12], and arthroscopic exploration [5,8] without resolution of symptoms. In all cases, the initial X-ray did not reveal any abnormalities, therefore subsequent CT [[6], [7], [8], [9], [10],^12^,^13^], MRI [5,[8], [9], [10], [11], [12]], bone scan [7,8], or Technetium-99 m [10,13] were done which ultimately revealed a clear nidus confirming the diagnosis of “OO”. Most cases underwent definitive treatment via an open surgical method [6,7,[9], [10], [11],^13^] whilst a minority was done via an arthroscopic approach [5,8,12].

Osteoid osteomas of the scapula are atypical, which is evident by the presence of a limited number of reported cases. This therefore results in misdiagnosis, as “OO” aren’t commonly thought of as a differential diagnosis in patients presenting with shoulder pain. According to Ogose et al., appropriate imaging modalities such as CT or MRI often visualize the characteristic nidus more clearly as opposed to an X-ray and should therefore be used early [15].

Treatment options include both surgical and non-surgical management. Surgical treatment can be achieved through complete excision of the nidus which is the treatment of choice. Two approaches for excision were suggested by Campanacci et al: wide en-bloc resection, and unroofing & excision [14]. En bloc resection involves the removal of the entire nidus -which is challenging in areas difficult to access-, whilst unroofing and curettage involves gradual removal of the overlying reactive bone, followed by excision with curettes [14]. This is preferred when the lesion is in a vital location.

Other options include percutaneous radiofrequency ablation which involves the destruction of the nidus by converting radiofrequencies into heat – a method followed by Rimondi et al. [16], or via arthroscopy which is indicated in cases of intra or juxta-articular “OO”s [14].

Our patient had a juxta-articular nidus affecting the base of coracoid process and therefore, arthroscopy was the preferred treatment of choice.

Based on our experience, having a high index of suspicion along with early use of advanced imaging modalities are essential for diagnosing such rare conditions. Therefore, we advise that young patients presenting with a long-standing history of shoulder pain not responding to conservative management should be investigated for osteoid osteomas.

## 4 Conclusion

Any young patient with ongoing, long-standing, non-traumatic shoulder pain that is worse at night and relieved by NSAIDs, whether it is associated with other features or not, should be investigated early with more advanced imaging modalities such as CT or MRI. This is essential to rule out the presence of a bone lesion which is better visualized by these modalities. These imaging modalities would allow earlier diagnosis of “OO” and therefore quicker treatment and subsequent quicker relief of the patients’ symptoms.

## Sources of funding

Nothing to declare.

## Ethical approval

Approval from the Bahrain Defence Force Royal Medical Services Research & Research Ethics Committee has been attained. Reference number: 2019-373.

## Consent

Written and signed consent from the patient has been obtained.

## Author contribution

- Dr. Dalal AlGhoozi (corresponding author) – writing the paper.
- Dr Hamza Gomaa (supervisor) – data collection.
- Dr Rashad Awad (supervisor) – data analysis.
- Dr Fahad Alkhalifa (supervisor) – study concept.

## Registration of research studies

Not applicable.

## Guarantor

Dr. Dalal AlGhoozi.

## Provenance and peer review

Not commissioned, externally peer-reviewed.

Written informed consent was obtained from the patient for publication of this case report and accompanying images. A copy of the written consent is available for review by the Editor-in-Chief of this journal on request.

## Declaration of Competing Interest

Nothing to declare.
